# Employee engagement mediates person-environment fit and new employees’ innovative behavior: A multidimensional perspective

**DOI:** 10.1371/journal.pone.0326161

**Published:** 2025-07-07

**Authors:** Liao Zeng, Shuai Song

**Affiliations:** 1 School of Economics, Chongqing Technology and Business University, Chongqing, China; 2 Business School, Southwest University of Political Science and Law, Chongqing, China; Southwest Petroleum University, CHINA

## Abstract

New employees can bring new perspectives and vitality to the organization. Creating a suitable environment for new employees to innovate, maintain their work enthusiasm, and stimulate their innovative behavior is an important research topic. This study focused on recent college graduates and included 893 valid samples collected from Chongqing, China. A structural equation model was constructed from a multidimensional perspective to explore the mediating role of employee engagement between person-environment fit and new employees’ innovative behavior. The results show that person organization fit, needs supplies fit, and demands abilities fit can directly increase new employees’ innovative behavior; Emotional and behavioral engagement mediate the relationship between person organization fit and needs supplies fit on innovative behavior, and behavioral and cognitive engagement mediate the relationship between demands abilities fit on innovative behavior. This provides practical suggestions for enterprise managers on effectively promoting employees’ innovative behavior and leading enterprises to realize sustainable development.

## Introduction

In today’s fast-changing business environment, innovation has become a core element for organizations to grow and maintain a competitive advantage. Employees’ innovative behavior is key to organizational performance, competitive advantage, and long-term survival [1]. Innovative behavior encompasses the generation of new products, technologies, ideas, and creativity, as referred to in the creation, promotion, and execution of these new ideas, focusing on realizing innovative ideas at a larger level [2]. Although innovative behavior is not typical of most employees’ work, it is seen as an extra-role behavior not directly driven by formal incentives or rewards [3]. However, employees’ innovative behavior remains critical in all industries [4]. Therefore, how organizations motivate their employees to innovate, how they create the right environment for them to innovate, and whether they can support and help their employees execute innovative ideas has become a key theme in organizational behavior research [5].

This study focuses on the innovative behavior of new employees, a group that plays an increasingly important role in the modern workplace. With the increasing proportion of Generation Z in the workplace, they are gradually becoming the mainstream of the workplace, and there are significant differences between them and the older generation of employees in terms of values, work, and lifestyles [6, 7]. This requires a deeper understanding of promoting innovative behavior in new employees by enhancing employee engagement. New employees’ innovative behavior is critical to organizational fit and growth and individual employees’ career development and satisfaction. Therefore, exploring new employees and person-environment fit and its impact on employee engagement and innovative behavior has essential theoretical and practical implications for organizations.

By examining the fit between individuals and their work environments, including person-organization fit and person-job fit, this study aims to reveal how these fits affect new employees’ emotional, behavioral, and cognitive engagement and innovative behavior and how different dimensions of employee engagement mediate these relationships. Through this study, we expect to provide strategic recommendations for organizations to stimulate new employees’ innovative potential and promote innovative organizational development.

### Person environment fit and new employees’ innovation behavior

Person environment fit (PEF) provides a unique perspective on how work environments motivate new employees’ innovation behavior (IB). Previous studies have more often examined the link between PEF and new employees’ job satisfaction, work attitudes, team performance, ability to adapt to a new organization, and employee turnover [8, 9]. As research on PEF becomes more in-depth, scholars pay more attention to new employees’ IB. PEF is a multidimensional concept that involves the degree of interaction and fit between the individual and the environment [10].

Individual organization fit refers to aligning an individual’s values, skills, and goals with those of the organization. Individuals perceive a strong match between themselves and the organization. They are more likely to experience a sense of belonging and commitment, which leads to increased motivation and intensity in innovative activities [11]. Social Exchange Theory states that individuals are more inclined to give back positively to an organization when they feel recognized, cared for, assisted, and respected by the organization [12]. The principle of reciprocity, which is central to Social Exchange Theory, emphasizes that individuals should give back to those who have helped them [13]. In the process of social exchange, resources are circulated through the mechanism of reciprocity, where the giver of positive behavior expects a positive response from the other [14]. Thus, when one party provides a valuable material or spiritual resource in social interactions, the beneficiary tends to reciprocate the resource giver with a corresponding return behavior. The benefits that employees derive from PEF help to satisfy their psychological needs, ensure the fulfillment of their personal values, and promote career development. As a result, when employees perceive encouragement and support, they are more likely to bring value to the organization by going the extra mile, such as giving back to the organization by demonstrating innovative behaviors [15].

Additionally, according to self-determination theory, employees who satisfy basic needs (e.g., when working with a sense of autonomy and control at work, possessing the skills needed for good performance, and working in a positive and supportive atmosphere) are more likely to pursue self-actualization, which includes innovative and creative work activities [16]. Empirical research suggests that PEF significantly promotes positive employee attitudes and behavioral performance [17]. In this area, Person organization fit (POF) and person job fit are the core embodiments of the concept of person environment fit, although other forms of employee fit properties exist in the workplace [18, 19]. POF and person job fit are important at all stages of an employee’s career, including the onboarding, permanent employment, and exit stages [20]. POF refers to the congruence between an employee’s values, personality, or goals and the work environment’s values, norms, or goals [21]. Person job fit is a combination of two job-related factors combinations of two job-related factors, including needs supplies fit (NSF) and demands abilities fit (DAF) [22, 23]. DAF describes the degree of fit between the demands of the work environment and the knowledge, skills, and attitudes that employees use to satisfy those demands [23]. In contrast, NSF reflects employees’ subjective evaluations of the organization’s provision of resources to meet their psychological needs [24]. Although innovation is an extra-role behavior for employees [25], organizations with higher POF tend to exhibit this behavior more frequently [26]. In addition, Goetz suggests that job-related expertise is one of the key factors influencing employee creativity and that the greater the fit between employees’ knowledge and skills in the workplace, the higher their level of IB [27]. That is, when employees’ abilities match the requirements of their jobs, they are more likely to feel confident and competent, which enhances their ability to solve problems and meet challenges. Competence and self-confidence are key drivers of innovative behavior because they motivate employees to try new approaches and take risks associated with innovation [28]. Afsar further confirmed the positive correlation between POF and a person’s job fit (including NSF and DAF) and an employee’s IB. That is, the employees who felt a high level of organizational Employees who are highly matched to the organization are more likely to engage in innovation [1]. Based on the above theoretical analysis and empirical research evidence, this study concludes that POF and person job fit positively contribute to employees’ IB. Therefore, we propose the following hypothesis:

*H1a: NSF is positively associated with new employees’* IB*.*

*H1b: DAF is positively associated with new employees’* IB*.*

*H1c: POF is positively associated with new employees’* IB*.*

### The role of employee engagement

Employee engagement is an individual’s simultaneous investment of body and mind endeavor to perform their work positively and fully [29]. It is widely accepted that employee engagement can increase an organization’s competitive advantage [30, 31]. Employee engagement is a state of full self-activation and an important mechanism for transforming inducements into organizationally desired employee attitudes and behaviors [32–34]. Empirical studies have emphasized that employee engagement is the basis of employees’ work attitudes and a key characteristic of positive work behavior. It encompasses three different types of inputs: emotional engagement (EE), behavioral engagement (BE), and cognitive engagement (CE) [35]. Employee engagement’s multifaceted nature distinguishes it from concepts such as job satisfaction, job engagement, and organizational commitment.

PEF is often considered a positive prerequisite for employee engagement [36]. Specifically, POF, NSF, and DAF influence employee engagement by shaping employees’ psychological state and behavioral performance, which affects employee engagement [37]. When POF is high, employees feel a stronger sense of belonging and identification, and this match also motivates employees at work, thus showing higher employee engagement [38, 39]. Furthermore, when employees’ values align with the organization and act authentically in their work environment, they perceive more autonomy, competence, and, as a result, more self-confidence, which positively contributes to their IB [40]. NSF focuses on the correspondence between employees’ personal needs and the resources provided by the organization. When employees feel that the organization can meet their development and growth needs, employee engagement and job satisfaction will significantly increase. On the other hand, needs-competence matching emphasizes the fit between employees’ skills and job requirements, and employee engagement and motivation are enhanced when employees feel that their competence is sufficient to cope with the challenges of the job [41].

Employee engagement is a core factor in creating a positive work environment, enhancing task performance, and stimulating key organizational outcomes such as IB [37, 42]. Research has shown that employee engagement significantly predicts their IB [37, 43–45], which stems from emotional engagement, behavioral engagement, and cognitive engagement synergy [46, 47]. Specifically, when employees are cognitively engaged in their work, they can revisit their experiences, broaden their horizons, and generate new ideas; affective engagement allows employees to be enthusiastic and optimistic about their work; and behavioral engagement is reflected in the employees’ proactive approach to challenges and difficulties, and their sustained investment of effort [41]. These input behaviors are closely related to the active participation of employees in innovation activities, thus promoting the innovation process within the organization [48]. Therefore, employee engagement reflects individual performance and significantly predicts organizational innovation capability. Based on the above analysis, we infer that POF, NSF, and DAF not only act directly on employee engagement but also can indirectly promote IB by enhancing employee engagement. Above all, we propose the following hypothesis:

*H2a: EE mediates the relationship between NSF and new employees’ IB.*

*H2b: EE mediates the relationship between DAF and new employees’ IB.*

*H2c: EE mediates the relationship between POF and new employees’ IB.*

*H3a: BE mediates the relationship between NSF and new employees’ IB.*

*H3b: BE mediates the relationship between DAF and new employees’ IB.*

*H3c: BE mediates the relationship between POF and new employees’ IB.*

*H4a: CE mediates the relationship between NSF and new employees’ IB.*

*H4b: CE mediates the relationship between DAF and new employees’ IB.*

*H4c: CE mediates the relationship between POF and new employees’ IB.*

## Materials and methods

### Ethics methods

This study was conducted by the Declaration of Helsinki and approved by the Ethics Committee of the Southwest University of Political Science and Law. We conducted the survey using an electronic questionnaire platform. The first page of the questionnaire was the informed consent form. Subjects were informed about the purpose of the study, the process, and data privacy. They were allowed to proceed with the questionnaire only if they clicked on consent to conduct the study.

### Participants

According to the definition of new employees in most previous empirical studies, the term “new” is used to refer to the length of time after the employee has been in the organization and employees who have worked in the organization for the first nine months or one year are referred to as new employees [49]. Therefore, the population of our study is recent college graduates who have been in the workplace for nine months to one year.

From March to April 2024, the research team sent recruitment advertisements to the QQ and WeChat contact groups of graduates with the help of counselors and career service teachers at three universities in Chongqing, China. At the same time, we recruited participants from 8 companies in Chongqing to include new employees willing to participate in this study. The first page of the e-questionnaire described the purpose of the study, the procedures, and the privacy of the participants, who had the right to withdraw from the study at any time. Informed consent was obtained from all subjects involved in the study. We promised that participants’ confidentiality was guaranteed throughout the survey. To encourage employees to participate actively in our survey, we randomly rewarded them with no more than 5 RMB after each completion of the questionnaire.

893 valid questionnaires were collected (questionnaires with missing values and logical errors were excluded, with a validity rate of 95.406%). The mean age of these was 24.141 (SD = 1.146) years. There were 477 (53.415%) male and 416 (46.585%) female participants. In terms of industry, participants were mainly from IT (20.941%), education (18.029%) and manufacturing (15.677%).

## Measures

### Person environment fit

This was measured using the Subjective Fit Perceptions Scale (SFPS), which consists of three dimensions, including POF, NSF, and DAF, each containing three items [22]. All items were measured on a 7-point Likert scale, where 1 = “strongly disagree” and 7 = “strongly agree.” In this study, the Cronbach’s α of this scale is 0.841.

### Employee engagement

The Employee Engagement Scale (EES) was used to measure employee engagement. The scale consists of 12 items and 3 dimensions (EE, BE, and CE), each including 4 items [50]. The scale was validated in the Chinese context [51]. A 5-point Likert scale was used for all the items, with 1 = “ strongly disagree” and 5=” strongly agree.” In this study, the Cronbach’s α of this scale is 0. 803.

### Innovative behavior

A 6 items Innovative Behavior Scale was used to measure new employees’ innovative behavior [52]. Sample items are“ Generates creative ideas “and” Promotes and champions ideas to others. “ The scale was validated in the Chinese context [53]. In this study, the Cronbach’s α of this scale is 0.855.

## Results

### Preliminary analysis

Table 1 shows that new employees who scored high levels of IB were more likely to have high levels of NSF (r = 0.566, p < 0.01), DAF (r = 0.504, p < 0.01), and POF (r = 0.451, p < 0.01). Similarly, new employees’ IB positively correlates with EE (r = 0.388, p < 0.01), BE (r = 0.500, p < 0.01), and CE (r = 0.488, p < 0.01).

**Table 1:** Descriptive Statistics and Correlation Analysis.

Variables	1	2	3	4	5	6	7
**1. Innovative Behavior**	1						
**2. Needs Supplies Fit**	0.566**	1					
**3. Demands Abilities Fit**	0.504**	0.590**	1				
**4. Person Organization Fit**	0.451**	0.632**	0.425**	1			
**5. Emotional Engagement**	0.388**	0.430**	0.300**	0.430**	1		
**6. Behavioral Engagement**	0.500**	0.514**	0.498**	0.451**	0.446**	1	
**7. Cognitive Engagement**	0.488**	0.341**	0.511**	0.248**	0.228**	0.402**	1
**Mean**	2.608	3.858	4.295	4.049	2.655	2.754	2.427
**SD**	0.679	0.841	0.854	0.882	0.702	0.774	0.767

Note: ** p < 0.01.

### Structural equation model analysis

Structural Equation Modeling (SEM) is a multivariate statistical analysis technique that combines the features of factor analysis and multivariate regression analysis. SEM allows the researcher to examine the relationships between multiple dependent and independent variables simultaneously [54]. The path coefficient indicates the relationship between the variables, and the relationship is significant when the p-value is less than 0.05. In H1abc~H4abc, we hypothesized the relationship between new employees’ PEF (3 dimensions), employee engagement (3 dimensions), and IB. We created a structural equation model (SEM) to test the above hypothesis. The fit indices showed the model fit well to the data (χ²/df = 2.885, CFI = 0.983, TLI = 0.964, GFI = 0.987, RMSEA = 0.046).

**Fig 1:**
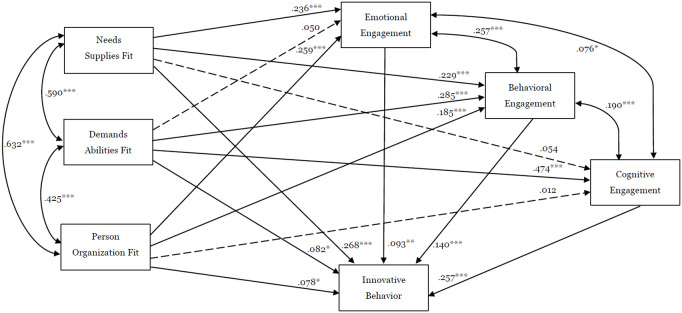
The result of the structural equation model (Standardized Estimate). Note: *** p < 0.001, ** p < 0.01, * p < 0.05; The dotted line means not significant.

The results of the SEM are shown in Table 2 and Fig 1. In the relationship between PEF and employee engagement, NSF (β = 0.197, P < 0.001) and POF (β = 0.206, P < 0.001) have a significant positive association with EE; NSF (β = 0.211, P < 0.001), DAF (β = 0.258, P < 0.001), and POF (β = 0.163, P < 0.001) all have positive associations with BE; Only DAF (β = 0.426, P < 0.001) has a significant associated with CE.

**Table 2:** The result of the structural equation model.

Path	B	β	S.E.	t	P
**NSF → EE**	0.197	0.236	0.036	5.537	<0.001
**DAF → EE**	0.041	0.050	0.030	1.364	0.173
**POF → EE**	0.206	0.259	0.030	6.809	<0.001
**NSF → BE**	0.211	0.229	0.036	5.812	<0.001
**DAF → BE**	0.258	0.285	0.031	8.441	<0.001
**POF → BE**	0.163	0.185	0.031	5.274	<0.001
**NSF → CE**	0.049	0.054	0.038	1.295	0.195
**DAF → CE**	0.426	0.474	0.032	13.280	<0.001
**POF → CE**	0.011	0.012	0.032	0.336	0.737
**EE → IB**	0.090	0.093	0.028	3.201	0.001
**BE → IB**	0.123	0.140	0.028	4.399	<0.001
**CE → IB**	0.227	0.257	0.026	8.773	<0.001
**NSF → IB**	0.216	0.268	0.030	7.202	<0.001
**DAF → IB**	0.065	0.082	0.027	2.397	0.017
**POF → IB**	0.060	0.078	0.025	2.375	0.018

Note: B: Unstandardized Estimate; β: Standardized Estimate.

Abbreviation: IB: Innovative Behavior; NSF: Needs Supplies Fit; DAF: Demands Abilities Fit; POF: Person Organization Fit; EE: Emotional Engagement; BE: Behavioral Engagement; CE: Cognitive Engagement.

Moreover, in the relationship between PEF and IB, NSF (β = 0.216, P < 0.001), DAF (β = 0.065, P < 0.05), and POF (β = 0.060, P < 0.05) all have positive associations with IB. Finally, in the relationship between employee engagement and IB, EE (β = 0.090, P < 0.01), BE (β = 0.123, P < 0.001), and CE (β = 0.227, P < 0.001) all have positive associations with IB as well.

### Effects test with bootstrap method

To avoid bias due to sample distribution, we tested the effect’s significance using the 5000-times Bootstrap method. The effect is significant when the 95% Bootstrap CI of the effect does not contain zero [55].

As shown in Table 3, the estimate of direct effect shows the significant effect between NSF (Effect = 0.055, BootCI= [0.028, 0.083]), DAF (Effect = 0.132, BootCI= [0.099, 0.176]), and POF (Effect = 0.041, BootCI= [0.015, 0.068]) with IB, H1a ~ H1c supported. The estimate of the indirect effect of EE shows that NSF (Effect = 0.018, BootCI= [0.005, 0.035]) and POF (Effect = 0.019, BootCI= [0.005, 0.037]) can influence IB via EE, H2a and H2c supported. The estimate of the indirect effect of BE shows that NSF (Effect = 0.026, BootCI= [0.013, 0.045]), DAF (Effect = 0.032, BootCI= [0.013, 0.055]), and POF (Effect = 0.020, BootCI= [0.008, 0.038]) can influence IB via BE, H3a~H3c supported. The estimate of the indirect effect of CE shows that only DAF (Effect = 0.097, BootCI= [0.069, 0.128]) can influence IB via CE, H4b supported.

**Table 3:** The result of testing the effects with the Bootstrap method.

Effect	Estimate	BootSE	Bootstrap 95% CI		P
			Lower	Upper	
**Total Effect**					
NSF → IB	0.271	0.040	0.188	0.351	0.002
DAF → IB	0.197	0.034	0.132	0.264	0.002
POF → IB	0.101	0.031	0.040	0.163	0.002
**Direct Effect**					
NSF → IB	0.055	0.014	0.028	0.083	0.002
DAF → IB	0.132	0.019	0.099	0.176	0.001
POF → IB	0.041	0.013	0.015	0.068	0.002
**Indirect Effect**					
NSF → EE → IB	0.018	0.008	0.005	0.035	0.009
DAF → EE → IB	0.004	0.003	−0.001	0.013	0.128
POF → EE → IB	0.019	0.008	0.005	0.037	0.010
NSF → BE → IB	0.026	0.008	0.013	0.045	0.001
DAF → BE → IB	0.032	0.010	0.013	0.055	0.001
POF → BE → IB	0.020	0.007	0.008	0.038	0.001
NSF → CE → IB	0.011	0.009	−0.005	0.029	0.154
DAF → CE → IB	0.097	0.015	0.069	0.128	0.001
POF → CE → IB	0.002	0.007	−0.011	0.018	0.694

Abbreviation: IB: Innovation Behavior; NSF: Needs Supplies Fit; DAF: Demands Abilities Fit; POF: Person Organization Fit; EE: Emotional Engagement; BE: Behavioral Engagement; CE: Cognitive Engagement

## Discussion

Innovation is critical to an organization’s continued growth in today’s business world. New employees can bring ideas and energy to a business and drive innovation. Many organizations hope to enhance development and innovation by recruiting new employees, often with poor results. The main problem is that companies neglect to match employees with the work environment and develop their innovation skills, especially regarding how to engage new employees in their work to promote innovation. Based on the social exchange theory, this study constructs the process of the influence of PEF on new employees’ IB from the perspective of person-environment interaction and the mechanism of employee engagement in this process. This study broadens the understanding of PEF, employee engagement, and new employee IB and clarifies their complex relationship from a more microscopic dimension.

First, the results of this study confirm that the three dimensions of PEF (NSF, DAF, and POF) are positively associated with new employees’ IB, H1a-H1c supported. This finding aligns with previous studies’ views that person-environment interaction in the workplace can affect the new employees’ IB [1, 56, 57]. This aligns with the connotation of person-environment fit, where compatibility between the person, the organization, and the work provides the motivational basis for completing work tasks. Firstly, person-organization fit mainly responds to whether the individual and the organization are compatible regarding values, culture, goals, and other directions. When the values of the organization and the individual are consistent, more employees can be inspired to produce more innovative behavior [58]. Secondly, DAF is also positively linked to the new employees’ IB. This result suggests that new employees judge their adaptability by comparing their abilities with the job demands. They are more inclined to display innovative behaviors when confident in coping with their jobs. This self-confidence not only raises their expectations for career advancement but also motivates them to improve their productivity and performance, increasing their willingness to take innovative actions [59].

The study’s results further revealed that the strongest positive correlation exists between NSF and new employees’ IB. This finding underscores the critical role of NSF in overcoming pressure and exhaustion in the innovation process, turning ideas into reality, and sustaining innovative motivation. [42]. In particular, NSF is achieved when organizations provide employees with intrinsic and extrinsic security or give the employees tangible career development opportunities and plans to drive new employees’ IB. From this point of view, the new generation of employees values improving their skills through training, has a pragmatic attitude, likes to take on challenges, and has high career expectations [60]. This also validates previous findings on the work values of China’s Generation Z employees that they may value career development, learning opportunities, and positive working relationships more than power, prestige, and corporate branding [61]. Consequently, when these needs, expectations, and developmental prospects are met through their work, employees who perceive NSF are also more likely to think optimally about solutions to problems and ultimately exhibit higher levels of innovation [62]. Thus, NSF plays a more significant role in influencing the new employees’ IB.

In addition, this study confirmed that the three dimensions of employee engagement (EE, BE, and CE) mediated the relationship between PEF and new employees’ IB. Specifically, EE played a mediating effect in the relationship between NSF, POF, and new employees’ IB, supporting hypotheses H2a and H2c; BE mediated the relationship between all dimensions of PEF and new employees’ IB, confirming hypotheses H3a through H3c; CE played a mediating effect in the relationship between DAF and new employees’ IB, supporting Hypothesis H4b. This finding reveals that POF and NSF shape employees’ IB primarily by influencing EE and BE, while DAF influences new employees’ IB primarily through BE and CE. The results suggest that POF and NSF are more strongly linked to IB by promoting employees’ affect and BE. This result can be explained by the fact that POF and NSF are more related to the alignment of employees’ values and goals with organizational values and resources and that these fit dimensions mainly influence employees’ positive affective and behavioral dimensions of engagement, which in turn promotes innovative thinking and creative problem-solving skills. This influence is directly linked to employees’ job satisfaction, organizational commitment, and work attitudes. When new employees are encouraged to behave authentically at work because their values are similar to those of the organization, they perceive themselves as more proactive and empowered and have a sense of organizational identity [40]. This promotes confidence in their future innovation efforts and optimism about the innovation process, helping them to inspire proactive behaviors throughout the organization [50, 63]. At the same time, these findings support Social Exchange Theory, which states that people seek fulfillment in exchange, whether material or non-material. For organizations to get their employees to contribute, they must provide incentives that drive them to put in more effort [26, 64]. It also highlights the importance of understanding employee motivation and its relationship to achieving organizational goals [13, 65]. However, because these fit dimensions are not directly related to the specifics of the job and the application of individual competencies, they have little impact on employees’ cognitive engagement, limiting their role in facilitating the mental dimension.

Finally, compared to POF and NSF, DAF significantly improves new employees’ IB mainly through behavioral and cognitive inputs. The core of DAF is to assess the match between employees’ knowledge and skills and the organization’s needs [22]. This boosts employees’ confidence in their job competence and promotes their behavioral and cognitive focus and engagement. In other words, when employees match their skills to the organization’s needs, they feel valued and give back to the organization practically [59]. The findings of this study not only enrich the theory of employee innovative behavior but also provide new perspectives on management practices. Adding new employees challenges the traditional management model, emphasizing the importance of matching between organizations, job design, and individual employees. In particular, DAF has a unique impact on enhancing employee engagement and innovative behavior. This suggests that modern managers need to emphasize new employees’ competency development, especially in knowledge acquisition and skill development. The latest generation of employees may be more in pursuit of personal growth and career development, and they want to acquire more knowledge and skills through their work to understand their roles and responsibilities better and to adapt their competencies to the requirements of their jobs.

## Implication

### Theoretical implication

This study reveals the direct effects of POF, NSF, and DAF on the new employees’ innovative behavior and the multidimensional mediating mechanism of employee engagement (EE, BE, and CE). The findings suggest that organizations need to pay systematic attention to the fit between the organization, job, and employee, which not only directly affects the innovative behavior of new employees but also plays an indirect role through different dimensions of employee engagement. Specifically, the significant effect of NSF suggests that organizations need to go beyond traditional compensation incentives to satisfy the diverse needs of employees through non-monetary support (e.g., career development opportunities, learning resources, psychological security, etc.) to stimulate their motivation to innovate. On the other hand, POF emphasizes the importance of values and cultural identity, and organizations can enhance employees’ sense of belonging by strengthening organizational culture, communication, and values alignment activities. In addition, DAF affects innovation through cognitive and behavioral engagement. Organizations should optimize the training system for new employees, especially in the early onboarding stages, to provide precise support for skill enhancement to increase their competency confidence, promoting innovative behaviors. These findings provide a theoretical basis for organizations to design more targeted management strategies and promote the transition from single incentives to multi-dimensional fit management models.

### Practical implication

This study provides important practical insights for organizations to cope with the challenges of managing new generation employees. First, organizations should build a demand-driven support system to accurately grasp the core demands of new employees (e.g., career development, job autonomy) through a dynamic demand identification mechanism (e.g., regular surveys, one-on-one communication), and design personalized support programs, such as mentorship or cross-departmental program participation, to strengthen the perception of NSF. Second, in terms of values integration and cultural adaptation, organizations need to communicate organizational values during the recruitment and onboarding stages, and use case studies, role modeling, and other methods to help new hires understand their fit with their personal goals, to enhance their willingness to innovate driven by POF. Third, to address the key role of DAF, organizations should establish a dynamic demand-ability assessment mechanism, combined with stage-by-stage skills training (e.g., digital tools workshops, innovation methodology courses) and real-world task assignments, to ensure that employees’ abilities match job requirements. Finally, based on the multi-dimensional mediating effect of employee engagement, organizations need to develop differentiated engagement activation strategies, such as enhancing EE by establishing a recognition culture, empowering challenging tasks to promote BE, and providing an innovative knowledge base to support CE, to form a complete chain of driving innovative behaviors. Particularly noteworthy is that the study warns organizations to avoid the problem of training lag, suggesting that learning resources (e.g., instant feedback tools) should be organically embedded into the daily workflow to meet the new generation’s special requirements for immediacy and adaptability. These practices effectively enhance organizational innovation output and help build a sustainable employee-organization symbiotic relationship, giving organizations an edge in the fierce competition for talent.

## Conclusion

Based on Social Exchange Theory, this study analyzes in depth the mechanism of PEF on new employees’ innovative behavior, with particular attention to the mediating effect of employee engagement. The results show that POF, NSF, and DAF can directly influence new employees’ innovative behavior, and needs-supplies fit has the most significant impact. In addition, POF and NSF mainly influence employees’ IB through the mediating role of EE and BE, while DAF mainly influences employees’ IB through the mediating role of BE and CE. In addition, POF and NSF primarily affect employees’ IB through the mediation of EE and BE, while DAF mainly affects employees’ IB through the mediation of BE and CE. These findings not only enrich the theoretical framework of employees’ innovative behaviors but also provide managers with practical guidance on how to promote innovative behaviors by enhancing the engagement of new employees.

### Limitation

The present study has limitations in several areas that must be further strengthened in future research. First, we used a cross-sectional design, which precludes making explicit causal inferences about the relationships between variables, though appropriate for our current study. We will attempt to use longitudinal data next to confirm our findings further. Second, our samples were all from China, limiting the findings’ breadth. In future studies, we will try to include new employees from more regions in the study. Finally, all variables were derived from new employees’ self-reports, and in future studies, we will consider including other relevant variables, such as business leaders’ style and coworkers’ support.

## Supporting information

S1 Dataset. (XLSX)
